# Clinical significance of immune cell infiltration within gallbladder cancer

**DOI:** 10.1038/sj.bjc.6601331

**Published:** 2003-10-28

**Authors:** Y Nakakubo, M Miyamoto, Y Cho, Y Hida, T Oshikiri, M Suzuoki, K Hiraoka, T Itoh, S Kondo, H Katoh

**Affiliations:** 1 Department of Surgical Oncology, Division of Cancer Medicine, Hokkaido University, Graduate School of Medicine, N-15 W-7, Kita-ku, Sapporo 060-8638, Japan; 2 Department of Pathology, Hokkaido University Hospital, N-14 W-5, Kita-ku, Sapporo 060-8648, Japan

**Keywords:** CD4, CD8, natural killer cells (NKCs), dendritic cells (DCs), gallbladder cancer, immunohistochemistry

## Abstract

To investigate the pathophysiological significance of infiltrating antitumour immune cells, we evaluated the quantity of immune cell intratumoral infiltration in 110 surgically resected gallbladder specimens by immunohistochemistry. We examined 45 cases of gallbladder cancer and 65 cases of benign gallbladder diseases for CD4^+^ T cells, CD8^+^ T cells, natural killer cells (NKCs), and dendritic cells (DCs). High levels of CD4^+^ T cell, CD8^+^ T cell, NKC, and DC infiltration were recognised in 51.1% (23 out of 45), 37.8% (17 out of 45), 33.3% (15 out of 45), and 48.9% (22 out of 45) of cancer specimens, respectively. High numbers of infiltrating CD4^+^ and CD8^+^ T cells correlated with decreasing tumour invasion, and high numbers of infiltrating DCs correlated with decreasing lymph-node tumour metastasis. Furthermore, increased infiltration of CD4^+^ and CD8^+^ T cells and DCs exhibited a significant correlation with prolonged survival. NKC infiltration, however, did not correlate with any of the clinicopathological factors examined. Additionally, high levels of infiltration were not identified in specimens from benign diseases, consistent with the cancer-specific activity of CD4^+^ and CD8^+^ T cells and DCs. In this study, we demonstrate that CD4^+^ and CD8^+^ tumour-infiltrating lymphocyte and DCs, but not NKCs, are important factors in the accurate prognosis of survival after surgical removal of gallbladder adenocarcinoma.

The prognosis for patients with gallbladder cancer remains poor despite improved diagnostic methods and surgical techniques. The 5-year survival rate after surgery is less than 15% (Donohue *et al*, 1990; Gall *et al*, 1991; Oertli *et al*, 1993; Cubertafond *et al*, 1994; Ruckert *et al*, 1996). The prognostic factors for gallbladder cancer are lymph-node metastases and the local extent of the primary tumour, which significantly influence surgical course and determine the prognosis after surgery (Tsukada *et al*, 1997; Sugawara *et al*, 1999). Recently, molecular biological studies have reported that specific cancer-derived proteins are associated with progression and prognosis in gallbladder cancer (Hui *et al*, 2000; Shi *et al*, 2000; Kawamoto *et al*, 2001; Quan *et al*, 2001).

Both tumour-specific and nonspecific lymphocytes play an important role in antitumour immune responses, through tumour recognition and immunological elimination of local and metastatic tumour cells (Boon *et al*, 1994; Brandle *et al*, 1996; Whiteside and Herberman, 1995; Tanaka *et al*, 1997).

A single malignant cell may have multiple tumour-specific antigens (Wortzel *et al*, 1983; Knuth *et al*, 1989; Van den Eynde *et al*, 1989). Cytotoxic CD8^+^ T cells recognise MHC class I molecules bearing antigenic peptides derived from endogenously synthesised proteins (Larsson *et al*, 2001). As the majority of tumours express MHC class I, CD8^+^ T cells can directly lyse tumour cells and destroy large tumour masses *in vivo* (Wang, 2001). In contrast to CD8^+^ T cells, CD4^+^ T cells recognise tumour-specific antigenic peptides on MHC class II molecules via an exogenous processing pathway (Larsson *et al*, 2001). Cytokines, such as IL-2 or IFN-gamma, released by CD4^+^ T cells upon antigenic stimulation are important for the antitumour effects of activated CD4^+^ T cells *in vivo* (Mumberg *et al*, 1999; Marzo *et al*, 2000; Beatty and Paterson, 2001). Activation of both T-cell subsets requires the presentation of antigenic peptides on professional antigen-presenting cells (APCs) (Larsson *et al*, 2001). Dendritic cells (DCs), the most potent APCs, play a central role in antitumour immunity by engulfing tumour antigens to facilitate the stimulation of antigen-specific T cells (Lespagnard *et al*, 1999; Almand *et al*, 2000). Natural killer cells (NKCs), comprising approximately 15% of all circulating lymphocytes, are a component of the innate immune system with the ability to lyse tumour cells and to provide early sources of immunoregulatory cytokines (Cooper *et al*, 2001).

Multiple immunohistochemical studies have described the relationship between immune-cell infiltration and tumour progression or prognosis in several cancers (Naito *et al*, 1998; Lespagnard *et al*, 1999; Ishigami *et al*, 2000a,2000b; Nakano *et al*, 2001; Schumacher *et al*, 2001). These studies have formed the basis for the practical application of immunotherapy (Malone *et al*, 2001; Valone *et al*, 2001; Ying *et al*, 2001; Barnea *et al*, 2002; Dhodapkar *et al*, 2002). The clinicopathological significance of infiltrating antitumour immune cells within gallbladder adenocarcinomas, however, has not been clarified.

We sought to determine the relationship of the intratumoural infiltration of CD4^+^ T cells, CD8^+^ T cells, NKCs, and DCs with tumour progression to identify favourable prognostic factors for patients with adenocarcinoma of the gallbladder.

## MATERIALS AND METHODS

### Patients

Between 1989 and 1999, we obtained samples from 110 gallbladders resected in the Department of Surgical Oncology, Division of Cancer Medicine at the Hokkaido University Graduate School of Medicine and affiliated hospitals. In every case, informed consent was obtained from the subject and guardian. In all, 45 cases were diagnosed as primary gallbladder adenocarcinoma (17 males and 28 females with a mean age of 66.7 years). No distant metastases were detected at preoperative examination. The clinical typing of tumours was performed according to the guidelines of the TNM Classification of Malignant Tumours (International Union Against Cancer, 1997). Nine tumours were classified as Stage I, 15 as Stage II, 5 as Stage III, and 16 as Stage IV.

Standard clinical treatment for advanced gallbladder cancer comprised removal of the gallbladder, wedge resection of the liver, resection of the extrahepatic bile duct, and resection of the regional lymph nodes. If the primary tumour or metastatic lymph nodes involve adjacent organs, including the head of the pancreas, duodenum, or colon, additional extended surgery to remove the affected area through pancreaticoduodenectomy, major hepatectomy, or colectomy was employed. Simple cholecystectomy was performed for early gallbladder cancer.

Benign diseases of the gallbladder included 20 cases of cholecystitis without anomalous pancreaticobiliary ductal junction (APBDJ), 24 cases of cholecystitis with APBDJ, 14 cases of adenomyomatosis, and seven adenomas. Among the seven gallbladder adenomas, six were diagnosed as tubular adenoma while one was characterized as a papillary adenoma. No evidence of intramucosal carcinoma was present in any of the adenoma cases. All cases of benign disease were treated by simple cholecystectomy.

Tissue specimens were fixed in 10% formalin and embedded in paraffin wax. Representative blocks including the greatest dimension of the tumour were selected; 4 *μ*m-thick serial sections were examined by immunohistochemistry.

### Immunohistochemistry

Immunohistochemical reactions were performed using the streptavidin-biotin-peroxidase method. The mouse monoclonal primary antibodies used were anti-human CD4, anti-human CD8 (pre-diluted, Nichirei Corporation, Tokyo, Japan), anti-human CD57 (Leu 7, Becton Dickinson Immunocytometry System, San Jose, CA, USA) at a dilution of 1 : 5, and anti S-100 protein (DAKO, Glostrup, Denmark) at a dilution of 1 : 2000. CD57 identifies cells originating from the neural crest, including NKCs (Sangueza and Requena, 1998; Ishigami *et al*, 2000a,2000b), while S-100 protein is an adequate marker of DCs (Lespagnard *et al*, 1999; Ishigami *et al*, 2000a). As positive controls, the normal adenoid tissue, prostate gland and nerve fibre were stained for CD4 and CD8, CD57, and S-100 protein, respectively. Sections were deparaffinised in xylene, washed in phosphate-buffered saline (PBS, pH 7.4), and rehydrated through a graded series of ethanol solutions. For CD4 and CD8 immunohistochemistry, following treatment with sodium citrate buffer (Ventana-Bio Tek Solutions, Tucson, AZ, USA), specimens were subjected to autoclave heat for 20 min, and to microwave heat for 20 min, respectively. Endogenous peroxidase activity was blocked by a 10 min incubation with 3% hydrogen peroxide methanol. Following washing in PBS, specimens were saturated with 10% normal goat serum (Histofine SAB-PO kit, Nichirei Corporation, Tokyo, Japan) for 5 min, then incubated at room temperature for 30 min with primary antibody. After washing in PBS, a biotinylated goat anti-mouse immunoglobulin antibody (Histofine SAB-PO kit, Nichirei Corporation, Tokyo, Japan) was applied during a 60 min incubation at room temperature. Immunohistochemical reactions were visualised with freshly prepared 3,3′-diamino-benzidine tetrahydrochloride (Histofine SAB-PO kit, Nichirei Corporation, Tokyo, Japan). Slides were counterstained with haematoxylin and mounted on coverslips. Immunostained sections were evaluated under a microscope (Olympus, Japan).

### Quantification methods

The degree of immune cell infiltration was observed in more than 10 independent high-power (× 200) microscopic fields (HPF) for each tissue sample. The three areas with the most abundant distribution were selected.

CD4^+^ and CD8^+^ T cells were quantified as described (Naito *et al*, 1998), with the following modifications. An average of more than 50 per three HPF accumulating CD4^+^ or CD8^+^ TILs were designated as infiltration. Individual cases were classified into high, low or no CD4^+^ or CD8^+^ TIL infiltration based on location and quantitation. The high group possessed immunoreactive T cells in the epithelial component of the tumour, either accumulating within the cancer cell nests and complexes or found predominantly in the mesenchymal stroma surrounding the epithelial component of the tumour. The low group exhibited immunoreactive T cells sparse, evenly distributed CD4^+^ or CD8^+^ TILs between the epithelial and stromal components and/or along the invasive margin of the tumour. The negative group did not demonstrate any immunoreactive T cells in either the epithelial and stromal components or along the invasive margin of the tumour.

Natural killer cells and DCs were quantified according to the method of Ishigami *et al* (2000a). In intratumoral fields, 10 or more NKCs per 10 HPF were defined as possessing high NKC infiltration, while less than 10 NKCs per 10 HPF were designated as low NKC infiltration. A total of 20 or more DCs per HPF were defined as high DC infiltration; less than 20 DCs per HPF characterised low DC infiltration.

In benign diseases, immunoreactive cells within and surrounding the epithelial lesion were quantitated.

The degree of immune cell infiltration for each section was represented by the median of scores evaluated by three investigators. All specimens were evaluated without any previous knowledge of the patients' clinical background.

### Statistical analysis

The *χ*^2^ test and Fisher's exact test were employed when appropriate. The Kaplan–Meier method was used to estimate the overall survival. Survival differences were analysed by the log-rank test, based on the status of immune cell infiltration. Univariate analyses of immune cell infiltration and clinicopathological features were performed using the Cox proportional hazards model. Probability (*P*) values of less than 0.05 were regarded as significant in all the analyses. Statistical analyses were performed using statistical software (StatView J version 5.0; Abacus Concepts Inc.).

## RESULTS

### Immunohistochemistry for immune cells

**Figure 1:**
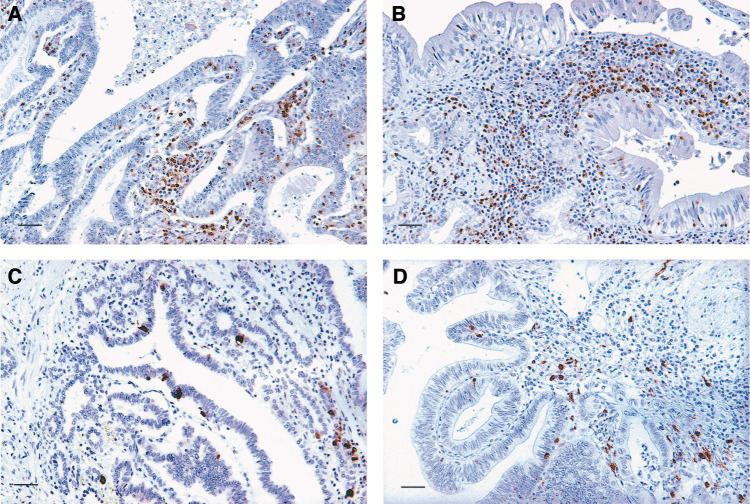
Representative photomicrographs of immunohistochemical staining of immune cells within gallbladder cancer (scale bar, 40 *μ*m). (**A**) high CD4^+^ TILs; (**B**) high CD8^+^ TILs; (**C**) high NKC infiltration; (**D**) high DC infiltration.

CD4^+^ T cells were distributed mainly along the invasive margin and in the cancer stroma. Of the total cancer cases, 51.1% (23 out of 45) were recognised as possessing high CD4^+^ T cell infiltration. In seven cases, TILs were identified within cancer cell nests (Figure 1A

). CD8^+^ T cells were also distributed along the invasive margin and within the cancer stroma; 37.8% (17 out of 45) possessed high CD8^+^ T cell infiltration. In 10 cases, TILs were found within cancer cell nests (Figure 1B). NKC infiltration varied from 0 to 7.3/HPF (average 1.2); 33.3% of the total cases (15 out of 45) contained high NKC infiltration. NKCs diffusely infiltrated the cancerous lesions (Figure 1C). DC infiltration varied from 0 to 88/HPF (average 22.8), with 48.9% (22 out of 45) exhibiting high DC infiltration. DCs were also observed diffusely throughout the lesions (Figure 1D).

**Figure 2:**
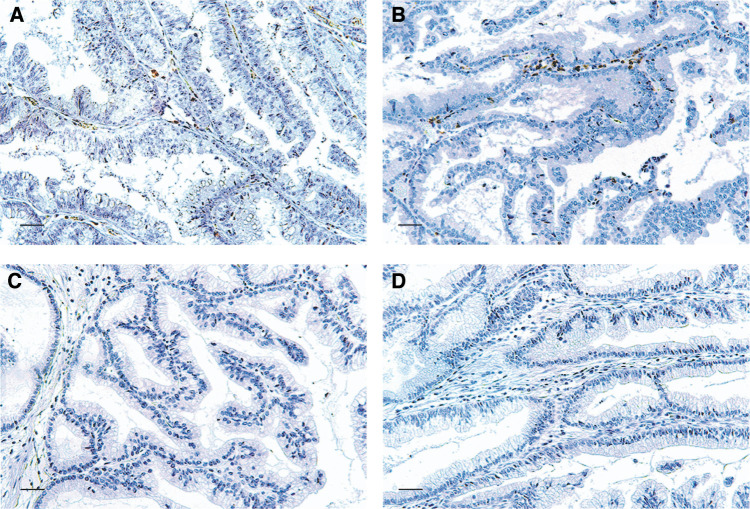
Representative photomicrographs of immunohistochemical staining of immune cells in adenomas of the gallbladder (scale bar, 40 *μ*m). (**A**) adenoma stained for CD4; (**B**) adenoma stained for CD8; (**C**) adenoma stained for CD57; (**D**) adenoma stained for S-100 protein.

In cases of benign disease, little or no infiltration of any kind of immune cells were observed (Figure 2

).

### Immune cell infiltration and clinicopathological significance

For the 45 patients with gallbladder cancer, we acquired pathological data on TNM classification, differentiation, vessel involvement, lymphatic involvement, perineural involvement, and stage grouping. Follow-up and clinical data were also obtained for all 45 patients. All cases of cancer evaluated in this study were diagnosed as adenocarcinomas. The details of high-level infiltration of immune cells and pT, pN, G categories, and p-stage grouping are indicated in Table 1

**Table 1:** High level infiltration of immune cells and histopathological findings in gallbladder cancer

**Variables**	**High CD4^+^ TILs (%)**	**High CD8^+^ TILs (%)**	**High NKC infiltration (%)**	**High DC infiltration (%)**
*Depth of invasion*				
pT1 (*n*=9)	5 (55.6)	5 (55.6)	3 (33.3)	6 (66.7)
pT2 (*n*=22)	16 (72.7)	11 (50.0)	7 (31.8)	12 (54.5)
pT3 (*n*=9)	1 (11.1)	1 (11.1)	4 (44.4)	2 (22.2)
pT4 (*n*=5)	1 (20.0)	0 (0.0)	1 (20.0)	2 (40.0)
				
*Lymph node metastasis*				
pN0 (*n*=27)	12 (44.4)	16 (59.3)	10 (37.0)	17 (63.0)
pN1 (*n*=6)	1 (16.7)	1 (16.7)	2 (33.3)	1 (16.7)
pN2 (*n*=12)	4 (33.3)	6 (50.0)	3 (25.0)	4 (33.3)
				
*Histopathological grading*				
G1 (*n*=27)	12 (44.4)	17 (63.0)	9 (33.3)	16 (59.3)
G2 (*n*=15)	3 (33.3)	4 (26.7)	6 (40.0)	4 (26.7)
G3 (*n*=3)	2 (66.7)	2 (66.7)	0 (0.0)	2 (66.7)
				
*P-stage grouping*				
I (*n*=9)	5 (55.6)	5 (55.6)	3 (33.3)	6 (66.7)
II (*n*=15)	11 (73.3)	7 (46.7)	6 (40.0)	10 (66.7)
III (*n*=5)	1 (20.0)	1 (20.0)	2 (40.0)	1 (20.0)
IV (*n*=16)	6 (37.5)	4 (25.0)	4 (25.0)	5 (31.3)

TILs=tumour-infiltrating lymphocytes; NKC=natural killer cell; DC=dendritic cell.

. No cases of pM1 were identified. The quantity of infiltrating CD4^+^ TILs correlated with vessel involvement, pT category, and stage grouping. The number of intratumoral CD8^+^ TILs correlated with age, vessel involvement, perineural involvement, and pT category. DC infiltration also correlated with pN category and stage grouping. NKC infiltration did not demonstrate any significant correlations with any of the examined clinicopathological factors (Tables 2

**Table 2:** Correlation between immune cells and clinicopathological features

		**CD4^+^ TILs**		**CD8^+^TILs**		**NKCs**		**DCs**	
**Variables**	**Total (*n*=45)**	**Low/high**	***P*^a^**	**Low/high**	***P*^a^**	**Low/high**	***P*^a^**	**Low/high**	***P*^a^**
*Age (years)*									
<69	22	14/8	0.0529	17/5	0.0417	16/6	0.3990	13/9	0.2949
⩾69	23	8/15		11/12		14/9		10/13	
									
*Sex*									
Male	17	7/10	0.4200	10/7	0.7141	14/3	0.0820	8/9	0.6718
Female	28	15/13		18/10		16/12		15/13	
									
*Vessel involvement*									
−	28	10/18	0.0233	14/14	0.0300	19/9	0.8279	12/16	0.1552
+	17	12/5		14/3		11/6		11/6	
									
*Lymphatic involvement*									
−	23	13/10	0.4578	12/11	0.1552	15/8	0.8330	12/11	0.8841
+	22	12/10		16/6		15/7		11/11	
									
*Perineural involvement*									
−	28	11/17	0.0981	14/14	0.0300	19/9	0.8279	12/16	0.1552
+	17	11/6		14/3		11/6		11/6	

a *χ*^2^ test. TILs=tumour-infiltrating lymphocytes; NKCs=natural killer cells; DCs=dendritic cells. The underlined numbers=significant.

, 3

**Table 3:** Correlation between immune cells and TNM classification, tumour differentiation, and stage grouping

		**CD4^+^TILS**		**CD8^+^TILs**		**NKCs**		**DCs**	
**Variables**	**Total (*n*=45)**	**Low/high**	***P*^a^**	**Low/high**	***P*^a^**	**Low/high**	***P*^a^**	**Low/high**	***P*^a^**
*pT*^b^									
1, 2	31	10/21	0.0009	16/15	0.0044	21/10	>0.9999^*^	13/18	0.0669
3, 4	14	12/2		13/1		9/5		10/4	
									
*pN*^c^									
0	27	11/16	0.1805	15/12	0.2586	17/10	0.5186	10/17	0.0207
1, 2	18	11/7		13/5		13/5		13/5	
									
*pM*^d^									
0	45	22/23	–	28/17	–	30/15	–	23/22	–
1	0	0/0		0/0		0/0		0/0	
									
*G*^e^									
1	27	10/17	0.0514	15/12	0.2586	18/9	>0.9999	11/16	0.0883
2, 3	18	12/6		13/5		12/6		12/6	
									
P-stage									
I, II	24	8/16	0.0256	12/12	0.0706	15/9	0.5262	8/16	0.0108
III, IV	21	14/7		16/5		15/6		15/6	

a *χ*^2^ test except for ^*^Fisher's exact test.

b depth of invasion;

c lymph node metastasis;

d distant metastasis;

e histopathological grading. TILs=tumour-infiltrating lymphocytes; NKCs=natural killer cells; DCs=dendritic cells. The underlined numbers=significant.

). Additionally, the strong correlation between TILs and depth of invasion is suggested in Table 1.

### Relationship among immunological factors

The number of infiltrating CD4^+^ cells also correlated with CD8^+^ TILs and DC infiltration (Table 4

**Table 4:** Correlation among immune cells

		**CD8^+^TILs**		**DCs**	
**Variables**	**Total (*n*=45)**	**Low/high**	***P*^a^**	**Low/high**	***P*^a^**
*CD4*^*+*^*TILs*					
Low	22	19/3	0.0011	17/5	0.0006
High	23	9/14		6/17	

a *χ*^2^ test. TILs=tumour-infiltrating lymphocytes; DCs=dendritic cells.

). There was no correlation, however, between the numbers of CD8^+^ TILs and DC infiltration (*P*=0.2989). NKC infiltration did not correlate with any other immunological factors.

### Correlation with survival

**Figure 3:**
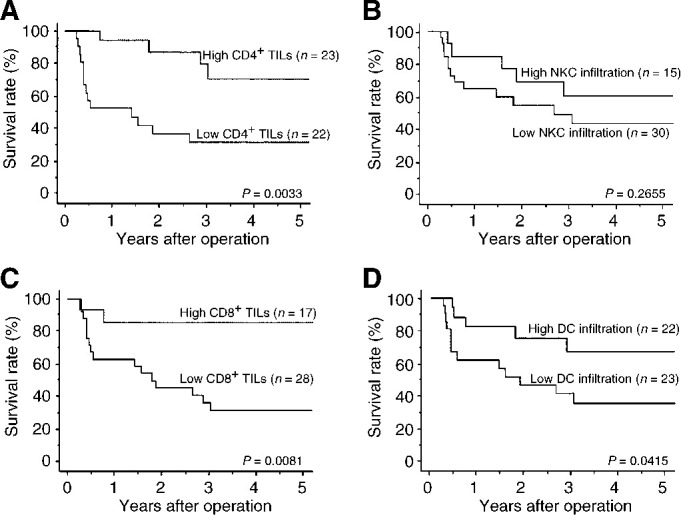
Kaplan–Meier analyses of the overall survival of gallbladder cancer patients with either a low or high level of immune cell infiltration. *P*-values were determined by the log-rank test. (**A**) CD4^+^ TILs; (**B**) CD8^+^ TILs; (**C**) NKC infiltration; (**D**) DC infiltration.

Using Kaplan–Meier actuarial analysis, the overall survival for patients possessing high numbers of CD4^+^ TILs, CD8^+^ TILs, and infiltrating DCs was significantly better than in exhibiting low infiltration (Figure 3A, B, and D

). NKC infiltration did not correlate with survival (Figure 3C).

The numbers of CD4^+^ and CD8^+^ TILs, age, pT category, pN category, G category, vessel involvement, lymphatic involvement, and perineural involvement were identified by Cox univariate regression analysis as significant prognostic predictors (Table 5

**Table 5:** Univariate analyses with Cox's proportional hazards model for imuune cells and clinicopathological features in all 45 patients

	**Univariate**		
**Variables**	**Hazards ratio**	**95% CI^a^**	***P*^b^**
CD4^+^TILs	0.218	0.071–0.666	0.0075
CD8^+^TILs	0.173	0.040–0.753	0.0194
NKCs	0.560	0.199–1.579	0.2727
DCs	0.358	0.128–1.006	0.0513
Age	0.215	0.076–0.609	0.0038
Sex	1.491	0.589–3.772	0.3990
pT	24.296	6.466–91.301	<0.0001
pN	9.651	3.499–26.619	<0.0001
G	5.419	1.989–14.760	0.0009
ly	5.518	2.022–15.060	0.0009
v	5.807	2.185–15.430	0.0004
ppn	4.733	1.831–12.237	0.0013

a CI=confidence interval;

b from Cox regression test. TILs tumour-infiltrating lymphocytes; NKCs=natural killer cells; DCs=dendritic cells. ly=lymphatic involvement; v=vessel involvement; ppn=perineural involvement.

).

## DISCUSSION

In gallbladder cancer, lymph-node metastases and depth of invasion of the primary tumour significantly affect patient outcome after surgery (Tsukada *et al*, 1997). Strong correlations between these pathological factors and the numbers of infiltrating immune cells suggest that the antitumour immune response may influence the prognosis of patients with gallbladder cancer in this study. The log-rank test revealed that high CD4^+^ TILs, high CD8^+^ TILs, and high DC infiltration are each individual favourable prognostic predictors.

Tumour-associated antigens (TAA) can be recognised by CD8^+^ T cells in the context of MHC class I-expressing tumours (Schumacher *et al*, 2001). Overall immune responses, however, were too weak and transient to eradicate cancer cells in the majority of patients receiving immunisation (Wang, 2001). Therefore, growing attention has been paid to the value of CD4^+^ T cells (Mumberg *et al*, 1999; Marzo *et al*, 2000; Beatty and Paterson, 2001; Wang, 2001) and DCs (Lespagnard *et al*, 1999; Almand *et al*, 2000) in antitumour immunity.

The correlation between DC infiltration and lymph-node metastasis observed in the present study conforms to the theory reported by Lespagnard *et al* (1999). Moreover, significant correlations were observed between CD4^+^ and CD8^+^ T cells and CD4^+^ T cells and DCs. These results support the mechanism of immune activation reported by Larsson *et al* (2001).

Compared with benign diseases, high levels of infiltrating CD4^+^ and CD8^+^ T cells and DCs were observed in cancer specimens. This outcome provides evidence that these immune cells possess cancer-specific activities in gallbladder cancer.

As NKCs were frequently identified in cancer specimens and not in benign diseases, NKC may have an ability to recognise cancer cells. NKC infiltration, however, did not correlate with either tumour progression or prognosis in this study. NKCs, constituting only a small portion of the tumour-infiltrating lymphocytes (Ishigami *et al*, 2000b), are components of the innate immune system capable of lysing target cells without prior sensitization (Cooper *et al*, 2001). Thus, the inability of NKCs to influence the outcome of disease may depend on the low proportion of total cells and weak anticancer specificity of NKCs.

We conclude as follows. Though the prognosis associated with TILs is not always good, CD4^+^ and CD8^+^ TILs and intratumoural DC infiltration, rather than NKCs, correlate with tumour progression, possibly serving as good prognostic predictors in patients with adenocarcinoma of the gallbladder.
